# Evaluation of the Abundance of DNA-Binding Transcription Factors in Prokaryotes

**DOI:** 10.3390/genes11010052

**Published:** 2020-01-03

**Authors:** Israel Sanchez, Rafael Hernandez-Guerrero, Paul Erick Mendez-Monroy, Mario Alberto Martinez-Nuñez, Jose Antonio Ibarra, Ernesto Pérez-Rueda

**Affiliations:** 1 Instituto de Investigaciones en Matemáticas Aplicadas y en Sistemas, Universidad Nacional Autónoma de México, Unidad Académica Yucatán, Mérida.C.P. 97302, Yucatán, Mexico; israel.sanchez@iimas.unam.mx (I.S.); rafaelhernandez291093@gmail.com (R.H.-G.); erick.mendez@iimas.unam.mx (P.E.M.-M.); 2 Unidad Académica de Ciencias y Tecnología de Yucatán, UMDI-Sisal. Facultad de Ciencias, UNAM, Mérida C.P. 97302, Yucatán, Mexico; maal.martinez@gmail.com; 3 Laboratorio de Genética Microbiana, Departamento de Microbiología, Escuela Nacional de Ciencias Biológicas, Instituto Politécnico Nacional, Ciudad de México C.P. 11340, Mexico; jaig19@gmail.com; 4 Centro de Genómica y Bioinformática, Facultad de Ciencias, Universidad Mayor, Santiago C.P. 7500000, Chile

**Keywords:** comparative genomics, transcription factors, protein families, prokaryote, genomes

## Abstract

The ability of bacteria and archaea to modulate metabolic process, defensive response, and pathogenic capabilities depend on their repertoire of genes and capacity to regulate the expression of them. Transcription factors (TFs) have fundamental roles in controlling these processes. TFs are proteins dedicated to favor and/or impede the activity of the RNA polymerase. In prokaryotes these proteins have been grouped into families that can be found in most of the different taxonomic divisions. In this work, the association between the expansion patterns of 111 protein regulatory families was systematically evaluated in 1351 non-redundant prokaryotic genomes. This analysis provides insights into the functional and evolutionary constraints imposed on different classes of regulatory factors in bacterial and archaeal organisms. Based on their distribution, we found a relationship between the contents of some TF families and genome size. For example, nine TF families that represent 43.7% of the complete collection of TFs are closely associated with genome size; i.e., in large genomes, members of these families are also abundant, but when a genome is small, such TF family sizes are decreased. In contrast, almost 102 families (56.3% of the collection) do not exhibit or show only a low correlation with the genome size, suggesting that a large proportion of duplication or gene loss events occur independently of the genome size and that various yet-unexplored questions about the evolution of these TF families remain. In addition, we identified a group of families that have a similar distribution pattern across *Bacteria* and *Archaea*, suggesting common functional and probable coevolution processes, and a group of families universally distributed among all the genomes. Finally, a specific association between the TF families and their additional domains was identified, suggesting that the families sense specific signals or make specific protein-protein contacts to achieve the regulatory roles.

## 1. Introduction

Gene regulation is crucial for optimal processes in the cell and as the first action to achieve expression during adaptation of metabolic responses to environmental conditions. In this context, regulation of gene expression at the transcriptional level, where DNA-binding transcription factors (TFs) play a fundamental role, allows the organisms to modulate the synthesis of specific genes depending on the metabolic requirements, stress responses, or food availability, among others. Hence, TFs interact with their DNA-binding sites around or overlapping the promoter-binding site [1,2], and in consequence allow or block access to the RNA polymerase, i.e. activating or repressing gene expression. In general, TFs are two-domain proteins, with a DNA-binding domain (DBD) in either the amino or carboxy terminus, which is involved in specific contacts with the regulatory region of the corresponding cognate genes, and an additional domain associated with diverse functions such as ligand binding or protein-protein interactions [3,4]. To date, diverse studies have shown that some TF families are common to bacteria and archaea, suggesting that the mechanisms affecting gene expression could be similar in the cellular domains of both of these groups of prokaryotes [5,6].

Therefore, a variety of factors are involved in the diversity of TFs and their families, such as the lifestyles. For instance, bacteria that have free-living lifestyles, such as *Pseudomonas aeruginosa* or *Escherichia coli*, bear a much larger number and variety of genes encoding transcriptional proteins than do intracellular pathogens that thrive in more stable biotopes [7,8]. In contrast, archaea organisms seem to have a lower proportion of TFs than bacteria, suggesting the existence in archaea of alternative mechanisms to compensate for the apparent deficit of protein regulators, including conformations of diverse protein complexes as a function of metabolic status [6,9].

Hence, to understand the association between the expansion patterns of different protein regulatory families, 1351 completely sequenced bacterial genomes, which represent adaptive designs for evolutionary classification, were analyzed. This analysis is important to understand their contribution to gene regulation in different lineages and provide insights into the functional and evolutionary constraints imposed on different classes of regulatory factors in bacterial and archaeal organisms. In this context, abundant families are not widely distributed across all bacteria and archaea. In contrast, certain small families are the most widely distributed. This difference might be associated with different phenomena, such as evolutionary constraints by regulatory mechanisms, as the case of LexA and LysR families. Our results also suggest that in larger genomes, regulatory complexity may possibly increase as a result of the increasing number of some TF families.

## 2. Materials and Methods 

### 2.1. Bacterial and Archaeal Genomes Analyzed

A total of 5321 prokaryotic genomes from the NCBI Refseq genome database [10] were downloaded, and to exclude any bias associated with the overrepresentation of bacterial or archaeal genomes of one genus or species, we employed a web-based tool and a genome similarity score (GCCa) of ≥0.95 [11] as the limit to consider a genome non-redundant; this method resulted in a set of 1321 representative genomes.

### 2.2. Identification of DNA-Binding Domains Associated with TFs

We retrieved 16,712 hidden Markov models (HMMs) from the PFAM database and used them to scan 5321 genomes, using the program pfam_scan.pl with an *E*-value of ≤10^−3^ and with the option of clan_overlap “activated” (to show overlapping hits within clan member families; this step only applies to Pfam-A families). In a posterior step, 111 PFAMs associated with DNA-binding TFs were retrieved from diverse databases containing information on regulatory proteins, such as the DBD (DNA-binding domain) database, Regulon Database, and Database of transcriptional regulation in *Bacillus subtilis* (DBTBs). We also identified relevant information by manual curation to identify proteins devoted to gene regulation. (The complete list of PFAM IDs is included as supplementary material Table S1).

### 2.3. Protein Domain Enrichment Analysis

To evaluate the content of protein domains associated with the 111 families of TFs, their structural domains were determined by considering the PFAM assignments and enrichment analysis for each group. To this end, we used a one-tailed Fisher’s exact test (FET) to perform enrichment analysis, because it is related to the hypergeometric probability and can be used to calculate the significance (*p*-value) of the overlap between two independent datasets. We set statistical significance at a *p*-value of −10. Together with the FET, we also determined the false discovery rate (FDR) of the tests in order to account for type I errors. Corrections for multiple testing were performed using the Benjamini and Hochberg step-up FDR-controlling procedure to calculate adjusted *p*-values. All analyses were performed using the R software environment for statistical computing and graphics and the package multtest [12].

## 3. Results

### 3.1. The Repertoires of TF Families Correlate with Genome Sizes

**Figure 1:**
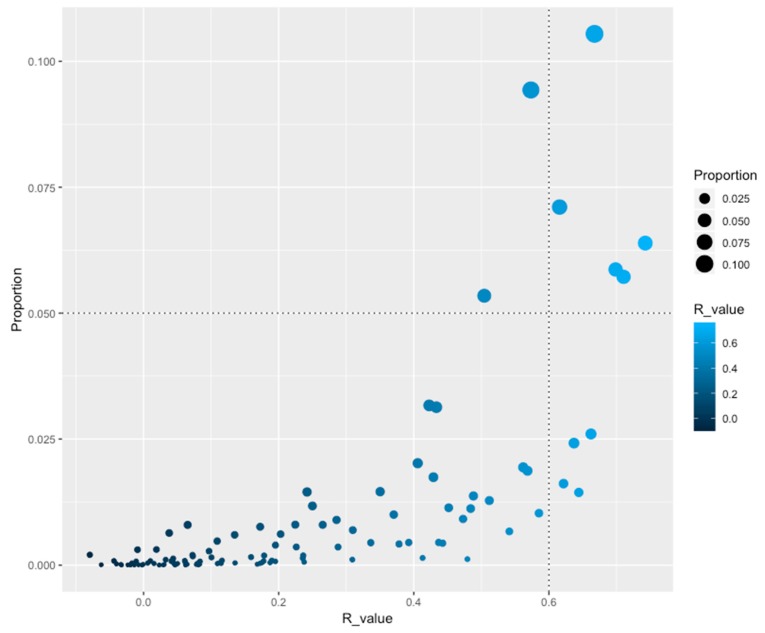
Proportion of TFs(Transcription factors) as a function of *R*-values. On the X-axis is the indicated the *R*-value (Pearson correlation) between the genome size and the number of proteins per family of TFs. On the Y-axis is the proportion of TFs versus the dataset. An *R*-value ≥ 0.6 was considered significant (vertical dotted lines at 0.6). *A* value ≥ 0.050 was considered as abundant (horizontal dotted lines). Circle size denotes the proportion of the families and color the *R*-value.

In order to identify how TF families are distributed as a function of genome size, the Pearson correlation (R-value) was calculated for the abundance of each family against the number of open reading frames (ORFs) associated with all genomes. From this, nine TF families were identified as correlating with genome size (*R* ≥ 0.6), such as the Trans_reg_C (PF00486) and GerE (PF00196) families, which include two-component systems, and seven families associated with a one-component system [GntR (PF00392), TetR_N (PF00440), MarR (PF01047), HxlR (PF01638), MerR_1 (PF13411), CSD, and HTH_AraC families] (Figure 1 and Table 1). These families followed a similar trend of duplication and loss events as a function of genome dynamics, reinforcing the notion that increased gene complexity also requires the development of mechanisms for gene regulation at the transcription level [13], i.e., when the genome is duplicated, members of these families are also duplicated, but when gene loss occurs these families are affected, decreasing the size of the family. An interesting observation of these families is the fact that they are regulating central functions in the organisms, such as carbon sources uptake (HTH_AraC) and resistance to multiple drugs, as antibiotics or heavy metals (MarR and MerR_1), among others. Conversely, 102 families did not exhibit an evident correlation with the genome size, suggesting that a large proportion of duplication or gene loss events occur independently from genome size, such as the highly abundant families LysR (PF00126) and HTH_3 (PF01381), associated to regulate amino acid biosynthesis and a hypothetical family widely distributed along the organisms.

### 3.2. The Abundance of Families is Not Homogeneous Across the Genomes

In order to determine how regulatory proteins abundant are along the prokaryotic genomes, a total of 225,999 TFs were identified in 1321 bacterial and archaeal genomes. These proteins were clustered into 111 different families, and their abundances and distributions along the genomes were evaluated. In this regard, some families are quite heterogeneous in terms of their abundances; for instance, 34 of these families include fewer than 100 proteins per group, such as the transcriptional repressor of *hyc* and *hyp* operons, HycA_repressor (PF11046), or the PerC transcriptional activator (PF06069), whereas two of them [HTH_1 (LysR) or PF00126, and TetR_N or PF00440] include more than 20,000 members per group. See Table 1. Therefore, to evaluate how the abundance of TF families correlated with the bacterial and archaeal genome sizes, we calculated the coefficient of variation (CV), a measure of the dispersion of data points in a data series around the mean. In this regard, large families showed a minor variation among the genomes, whereas small families exhibited a wide variation (presence and abundance) among the organisms analyzed in this work. For instance, the large families Trans_reg_C (PF00486), with 14,446 members, exhibits an average 10.7 ± 10.91 proteins per genome (CV = 1.01); GntR (PF00392), with 13263 members, has 9.90 proteins per genome (CV = 1.33), and TetR_N (PF00440) had 12.02 proteins per genome (CV = 2.6). In contrast, the Histone_HNS family (PF00816) has 434 members (CV = 3.67), the AP2 (PF00847) family has 77 members (CV = 5.49), and the CitT (PF12431) family has 82 proteins (CV = 5.05). These results suggest that small families, such as AP2 (PF00847), CitT, or Histone_HNS, have a heterogeneous distribution and abundance among bacteria and archaeal genomes, whereas large families have a small CV, i.e. they have a more homogeneous distribution of size among the genomes. See Table 1.

**Figure 2:**
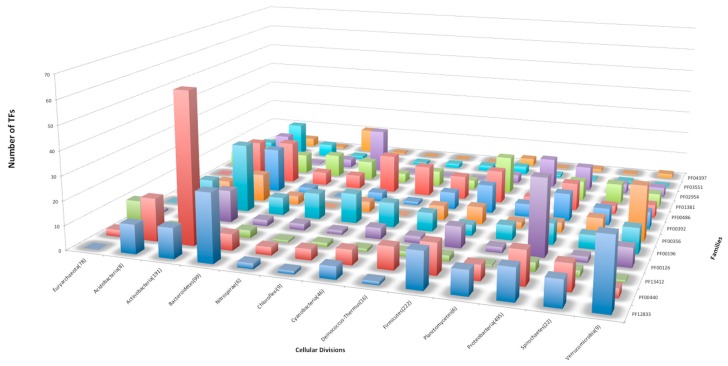
Families identified with more than 10 members (on average) per cellular division. The Y-axis shows the number of TFs, and the X- and Z-axes indicate the cellular division and family ID, respectively.

When the abundance of the families was analyzed in detail, 12 of them were identified with more than 10 members per phylum (on average), whereas the rest of the families contained a low number of copies per phylum. In Figure 2 we show that seven families with more than 10 members were identified in *Actinobacteria*, 6 in *Proteobacteria* and *Acidobacteria*, and 5 are abundant in Firmicutes. There are three families in *Verrumicrobia,* two families each in *Chlorobi, Cyanobacteria, Nitrospora, Planctomyces,* and *Bacterioidete.* The family HTH_24 [or AsnC (PF13412)] was found to be abundant in *Euryarchaeota*, and the family Trans_reg_C (PF00486) is abundant in *Deinococcus*. Indeed, the family AsnC (HTH_24), abundant in Euryarchaeota, has been described as a group with global regulators in bacteria and archaea, suggesting a role in global regulation [14].

### 3.3. Correlation of TF Families among Bacteria and Archaeal Genomes

In order to evaluate if the families show a common distribution pattern that would allow us to hypothesize about potential correlation patterns and in consequence families working together, we calculated their coefficient of correlation, and a matrix of ALL *versus* ALL was created. From this matrix, a hierarchical cluster (HCL) analysis was achieved with a Manhattan distance and support tree with average linkage algorithm, with correlation uncentered as a similarity measure [15]. From this analysis, a total of 90 families were included in 15 clusters, whereas 21 families were not included in an evident cluster. Therefore, the clustering of families with similar distribution patterns suggests the existence of common distribution patterns as a consequence of regulation via similar mechanisms, such as the cluster in which members of the YoeB_toxin (PF06769), PhdYeFM_antitox (PF02604), and ParE_toxin (PF05016) families, all of which belong to toxin-antitoxin systems and are associated with the clan CL0136, were included. Indeed, proteins of these families interact among themselves to regulate postsegregation cell killing systems that might function as regulatory switches under stress conditions [16], and are involved in initiate cell death in bacterial and archaeal cultures and to content against the infection by phages or to regulate subpopulations [17].

Another interesting cluster is integrated by the flagellar regulation families (PF05247 FlhD and PF05280 FlhC); histone-like proteins such as Histone_HNS (PF00816); the Phage_AlpA (PF05930), BolA (PF01722), and Arc (PF03869) familes; and the Pro_dh-DNA_bdg (PF14850) family. All these familes exhibit a similar correlation pattern of distribution. In this regard, proteins of the bacterial flagellar transcriptional activator (FlhC) combine with FlhD to form a regulatory complex in *E. coli* [18] or members of the histone-like nucleoid-structuring (H-NS) protein, which plays a role in the formation of nucleoid structure. In addition, AlpA is in a family that consists of several short bacterial and phage proteins that are related to the *E. coli* protein AlpA, whereas BolA causes round morphology and may be involved in switching the cell between elongation and septation systems during cell division [19]. It has also been suggested that BolA induces the transcription of penicillin-binding proteins 6 and 5 [20]. In summary, these findings suggest that families work together to regulate common functions in bacteria and archaeal genomes, such as the FlhD and FlhC families, or AlpA and BolA families, and opens diverse correlations to be further analyzed in functional and structural terms to identify potential protein-protein contacts or similar regulatory mechanisms. 

### 3.4. Distributions of Families among All the Genomes

**Figure 3:**
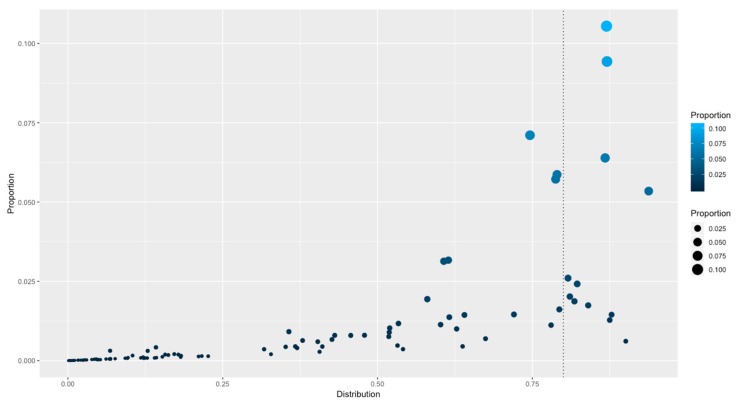
Distribution of TF families among all the genomes. The X-axis indicates the distribution (as a percentage) of genomes with members of a given family. 0 represents absence of a family and 1 means that a family is distributed along all the organisms. The Y-axis indicates the proportion of families versus the total of TFs identified. The vertical dotted line indicates those families identified in more than 80% of organisms.

To determine how the families are distributed among the complete collection of bacterial and archaeal genomes and to determine if there are families that are universally distributed, the distributions of the 111 PFAMs were traced along the 1321 genomes, and their rates of occurrence were calculated, considering the rate of total presence of a PFAM against the total number of organisms. Therefore, a value close to 1 indicates that the family is present in 100% of the organisms, whereas a value near 0 indicates that the family is absent in all the organisms. Table 1 and Figure 3. From this distribution, a set of 12 families were considered universally distributed, because they were found as in at least 80% of the total organisms, and they could be considered the basic core of regulators associated with prokaryotes. In this dataset, the following families were identified: HTH_3 (PF01381), Bac_DnaA_C (PF08299), Bac_DNA_binding (PF00216), Fur (PF01475), HTH_5 (PF01022), MerR_1 (PF13411), HTH_Crp_2 (PF13545), HTH_24 (PF13412), MarR (PF01047) and TetR_N (PF00440), HTH_1 (PF00126), and Trans_reg_C (PF00486). The rest of the families identified in all the genomes can be interchanged or lost among the bacteria and archaea as a consequence of their lifestyles. In general, the set of universal families is comprised of highly abundant groups, as TetR (highly abundant) and those that are not necessarily the most abundant ones, suggesting that families with a small number of members are also fundamental for the regulation of basic processes, such as ferric uptake regulator Fur, that connects iron transport and utilization enzymes with negative-feedback loop pairs for iron homeostasis [21]; Fur has been identified across a large diversity of organisms [22,23,24]. Similarly, DnaA is involved in initiation of chromosomal replication [25], a fundamental process of all organisms; or LexA-like proteins, with a wide distribution along the bacteria and archaeal genomes, suggesting that the SOS response might be a universal adaptation of bacteria to DNA damage [26]. This distribution, together with the probable coevolution of the LexA recognition domain and its binding motif [27], indicates that the regulated genes must also contain a conserved binding motif in the upstream regions. In summary, we suggest a probable scenario concerning the distribution of universal or widely distributed families, with a conservation of the regulated genes such as the SOS regulon and LexA, with few probable recruitments of additional TFs to regulated regulons, such as occurs in the evolution of regulatory networks [28].

### 3.5. Structural Domains Associated with Families

**Figure 4:**
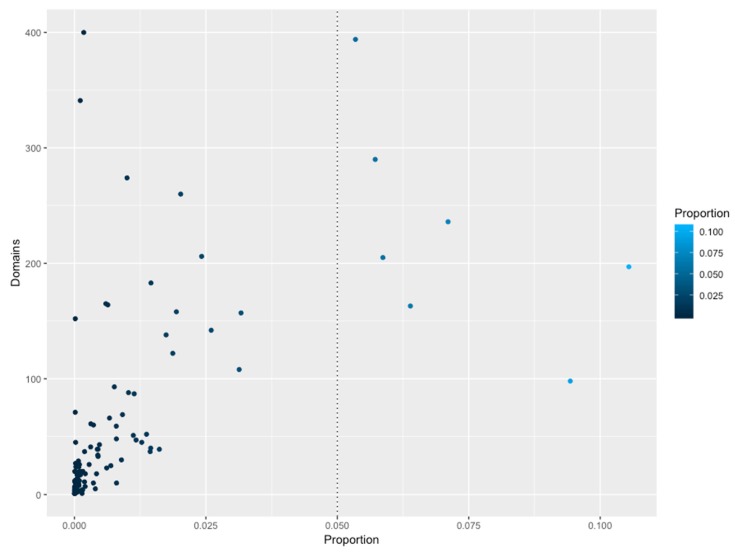
Number of NR domains associated with TF families. The X-axis indicates the proportion (as a percentage) of abundance of a given family versus the total of TFs. The Y-axis indicates the total number of non-redundant domains per family. The dotted line indicates the most abundant families.

**Figure 5:**
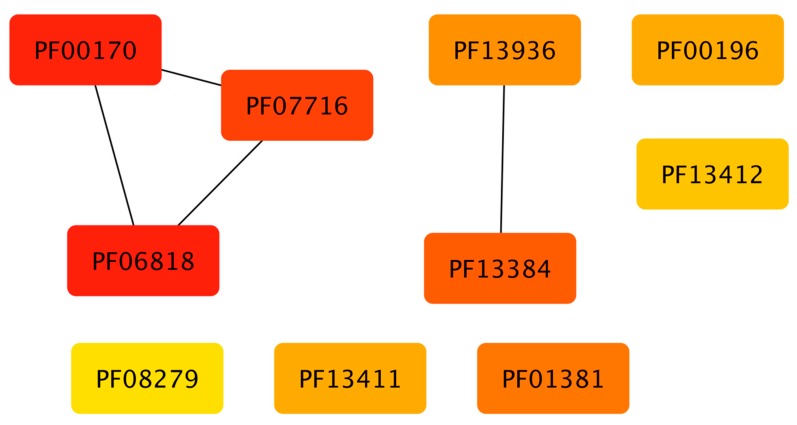
Top 10 families of TFs identified by MCC(Maximal Clique Centrality). Each box represents a protein family (see Section 3.5) and lines between boxes indicate that some families share additional domains.

To gain insights into the association between the structural domains connected with the families, these groups of proteins were analyzed in terms of their domains. In total, 2694 different domains were identified to be associated 111 families. In Figure 4, the association between those domains and families, shows that abundant families do not have a high diversity of structural domains, i.e. small families, constrained to specific phyla, contain a large proportion of additional domains, such as the Fez1 with few members associated to more than 300 different domains. Similarly, large and universal families also contain a large number of domains, such as the Trans_reg_C with around 160. Therefore, to determine if there is a specific association between the domains identified and the families, an enrichment analysis using a one-tailed FET was performed, and a significance of *p*-value of less than −10 was considered. Table 1 shows the number of domains identified in all the families, and in Figure 5 (and supplementary material S1), a network representation of all associations between TFs and additional domains (enriched domains) is shown. From this network representation, we evaluated the most important nodes (TF families) by using the Maximal Clique Centrality (MCC) method, because it has been described to show excellent performance and precision in predicting essential proteins from networks [29]. Based on this approach, we identified a set of 10 families as highly important and specifically connected with their respective domains, including Fez1 (PF06818), bZIP_1 (PF00170), and bZIP_2 (PF07716), sharing domains among them, and suggesting that they could be associated with similar regulatory processes. In contrast, HTH_3 (PF01381) is strongly associated with the Methyltransf_22 (PF13383), suggesting that one of the most common architectures of this family contains those domains. In addition, the HTH_23 (PF13384), HTH_38 (PF13936), GerE (PF00196), MerR_1 (PF13411), HTH_24 (PF13412), and HTH_11 (PF08279) are described in the dataset as regulators with a large diversity of domains (more than 60 different domains), and are not linked among them, suggesting that they contain specific domains with few domains that are shared with other families of TFs. Therefore, we suggest that combinations between the DNA-binding domains and their associated domains significantly increase the sensing of diverse signal compounds, decreasing signaling cross talk and making the response to environmental stimuli in bacterial and archaeal organisms more efficient.

## 4. Discussions

In this work, we evaluated 111 families of DNA-binding TFs on bacterial and archaeal genomes, how abundant they are in prokaryotic genomes, and how they are distributed according to genome size. For the examined families, we found a set of nine families whose distribution correlates with the genome size and that represent more than 40% of the total of TF identified by HMM profiles. These families have been intimately associated with diverse and central functions in the organisms, such as the two-component systems (Trans_reg_C and GerE), multiple antibiotic resistance responses (TetR_N and MarR), or carbon sources uptake, virulence, and nitrogen assimilation (HTH_AraC), among others. The correlation between the abundance of these families and the genome size reinforces the notion that increased gene complexity also requires the development of mechanisms for gene regulation at the transcription level [13]. In contrast, 56.3% of the collection do not exhibit a clear correlation with the genome size, suggesting that a large proportion of families have independent evolutionary events associated with their increasing, such as duplications or gene losses, opening questions to be further explored, such as how many families in a genome are product of lateral gene transfer or what occurs with the regulated genes or how many families with a similar distribution pattern across *Bacteria* and *Archaea*, are product coevolution processes. In addition, we found a specific association between the DNA-binding domains and their associated companion domains, as it has previously described [3], suggesting that the scaffold to protein-protein interactions could be conserved among members of the same family contacts, as occurs in the Crp family and that their association in diverse bacterial and archaeal genomes could increase the ability of the organisms to recognize and respond to diverse environmental stimuli [30]. This result opens the opportunity to predict and modify the probable ligands to understand the diversity of signals that modulate the activity of transcription factors, as it has been identified for *E. coli* [31].

Finally, based on a correlation matrix of all families, we identified a probable coevolution processes of families devoted to regulate similar processes, such as the members of the YoeB_toxin (PF06769), PhdYeFM_antitox (PF02604), and ParE_toxin (PF05016) families exhibited similar distribution patterns among all the bacterial and archaeal genomes, suggesting that the regulation to initiate cell death in bacterial and archaeal cultures is widely distributed to content against the infection by phages or to regulate subpopulations [17].

## 5. Conclusions

In conclusion, diverse scenarios might occur, depending notably on the family of TF associated, such as those abundant and universal families devoted to regulate amino acid biosynthesis (LysR) or antibiotic resistance (TetR and MarR), or those less abundant ones such as the LexA family, whose distribution suggest that the SOS response might be a universal adaptation of prokaryotic organisms to DNA damage [26].

## Figures and Tables

**Table 1:** Families evaluated in this work. Columns are as follows: PFAM ID, PFAM Name and PFAM Description: *R*-value (correlation between genome size and PFAM distribution); Total number of members in the bacterial and archaeal genomes; Proportion of the PFAM (%) relative to the complete collection; PFAM Coefficient of Variation (CV); Number of total of domains; Number of different domains (NR); PFAM Distribution (%) among all the organisms.

PFAM ID	PFAM Name	PFAM Description	Correlation (*R* value)	Total No. of Proteins	Proportion	CV	Domains (Total)	Domains (NR)	Genomic Distribution (%)
PF00096	zf-C2H2	Zinc finger, C2H2 type	−0.04	59	0.00	6.82	105	24	0.03
PF00126	HTH_1	Bacterial regulatory helix-turn-helix protein, lysR family	0.57	21320	0.09	1.70	42400	98	0.87
PF00165	HTH_AraC	Bacterial regulatory helix-turn-helix proteins, AraC family	0.05	47	0.00	6.85	86	27	0.03
PF00170	bZIP_1	bZIP transcription factor	0.03	248	0.00	3.11	4587	341	0.12
PF00196	GerE	Bacterial regulatory proteins, luxR family	0.71	12932	0.06	1.53	25020	290	0.79
PF00216	Bac_DNA_binding	Bacterial DNA-binding protein	0.24	3276	0.01	0.95	4731	40	0.88
PF00313	CSD	’Cold-shock’ DNA-binding domain	0.62	3651	0.02	0.91	3866	39	0.79
PF00325	Crp	Bacterial regulatory proteins, crp family	0.18	186	0.00	2.70	377	24	0.13
PF00356	LacI	Bacterial regulatory proteins, lacI family	0.43	7083	0.03	1.71	14525	108	0.61
PF00376	MerR	MerR family regulatory protein	0.54	1506	0.01	1.65	2885	66	0.43
PF00392	GntR	Bacterial regulatory proteins, gntR family	0.70	13263	0.06	1.33	25478	205	0.79
PF00440	TetR_N	Bacterial regulatory proteins, tetR family	0.67	23839	0.11	2.62	33009	197	0.87
PF00486	Trans_reg_C	Transcriptional regulatory protein, C terminal	0.74	14446	0.06	1.01	31282	163	0.87
PF00816	Histone_HNS	H-NS histone family	0.24	434	0.00	3.68	537	37	0.16
PF00847	AP2	AP2 domain	0.08	77	0.00	5.50	105	13	0.04
PF01022	HTH_5	Bacterial regulatory protein, arsR family	0.43	3940	0.02	0.93	4683	138	0.84
PF01047	MarR	MarR family	0.66	5880	0.03	1.15	6384	142	0.81
PF01258	zf-dskA_traR	Prokaryotic dksA/traR C4-type zinc finger	0.31	1564	0.01	1.02	1744	25	0.67
PF01316	Arg_repressor	Arginine repressor, DNA binding domain	0.10	631	0.00	1.33	1287	26	0.41
PF01325	Fe_dep_repress	Iron dependent repressor, N-terminal DNA binding domain	0.11	1076	0.00	1.23	2675	43	0.53
PF01340	MetJ	Met Apo-repressor, MetJ	0.05	72	0.00	4.27	73	2	0.05
PF01371	Trp_repressor	Trp repressor protein	0.04	299	0.00	2.00	330	20	0.21
PF01381	HTH_3	Helix-turn-helix	0.50	12084	0.05	0.82	16741	394	0.94
PF01402	RHH_1	Ribbon-helix-helix protein, copG family	0.17	1714	0.01	1.56	2228	93	0.52
PF01418	HTH_6	Helix-turn-helix domain, rpiR family	0.27	1802	0.01	1.74	3574	48	0.43
PF01475	FUR	Ferric uptake regulator family	0.51	2891	0.01	0.64	2992	45	0.87
PF01638	HxlR	HxlR-like helix-turn-helix	0.64	3255	0.01	1.65	3389	37	0.64
PF01722	BolA	BolA-like protein	0.20	898	0.00	1.36	905	5	0.37
PF01726	LexA_DNA_bind	LexA DNA binding domain	0.44	1015	0.00	0.91	1988	33	0.64
PF01978	TrmB	Sugar-specific transcriptional regulator TrmB	0.04	1436	0.01	2.47	2475	164	0.38
PF02082	Rrf2	Transcriptional regulator	0.48	2531	0.01	0.85	2644	51	0.78
PF02257	RFX_DNA_binding	RFX DNA-binding domain	0.03	12	0.00	13.56	28	7	0.01
PF02467	Whib	Transcription factor WhiB	0.38	947	0.00	2.92	1000	18	0.14
PF02604	PhdYeFM_antitox	Antitoxin Phd_YefM, type II toxin-antitoxin system	0.29	2020	0.01	1.58	2104	30	0.52
PF02892	zf-BED	BED zinc finger	−0.01	17	0.00	11.11	30	11	0.01
PF02954	HTH_8	Bacterial regulatory protein, Fis family	0.42	7162	0.03	1.51	20550	157	0.61
PF03333	PapB	Adhesin biosynthesis transcription regulatory protein	0.05	14	0.00	11.01	22	5	0.01
PF03551	PadR	Transcriptional regulator PadR-like family	0.49	3098	0.01	1.62	3782	52	0.62
PF03749	SfsA	Sugar fermentation stimulation protein	0.07	458	0.00	1.46	475	7	0.33
PF03869	Arc	Arc-like DNA binding domain	0.20	171	0.00	3.50	174	3	0.10
PF03965	Penicillinase_R	Penicillinase repressor	0.39	1013	0.00	1.88	1081	39	0.37
PF04014	MazE_antitoxin	Antidote-toxin recognition MazE, bacterial antitoxin	0.07	1798	0.01	1.72	2321	59	0.46
PF04024	PspC	PspC domain	0.34	1004	0.00	1.47	1300	34	0.41
PF04221	RelB	RelB antitoxin	-0.08	466	0.00	2.96	499	18	0.17
PF04247	SirB	Invasion gene expression up-regulator, SirB	0.12	213	0.00	2.56	291	26	0.14
PF04299	FMN_bind_2	Putative FMN-binding domain	0.41	325	0.00	2.04	325	1	0.22
PF04353	Rsd_AlgQ	Regulator of RNA polymerase sigma(70) subunit, Rsd/AlgQ	0.11	98	0.00	3.74	112	4	0.07
PF04383	KilA-N	KilA-N domain	0.06	136	0.00	4.19	160	14	0.08
PF04397	LytTR	LytTr DNA-binding domain	0.25	2649	0.01	2.13	4836	47	0.53
PF04606	Ogr_Delta	Ogr/Delta-like zinc finger	0.11	80	0.00	4.94	94	10	0.05
PF04761	Phage_Treg	Lactococcus bacteriophage putative transcription regulator	−0.01	1	0.00	36.47	1	1	0.00
PF04947	Pox_VLTF3	Poxvirus Late Transcription Factor VLTF3 like	−0.02	14	0.00	12.86	25	12	0.01
PF04967	HTH_10	HTH DNA binding domain	0.02	704	0.00	7.47	1610	61	0.07
PF05016	ParE_toxin	ParE toxin of type II toxin-antitoxin system, parDE	0.22	1808	0.01	1.63	1839	10	0.48
PF05043	Mga	Mga helix-turn-helix domain	−0.01	695	0.00	5.32	2544	41	0.13
PF05068	MtlR	Mannitol repressor	0.06	37	0.00	6.54	38	2	0.02
PF05225	HTH_psq	helix-turn-helix, Psq domain	0.08	50	0.00	6.45	139	45	0.03
PF05247	FlhD	Flagellar transcriptional activator (FlhD)	0.18	114	0.00	4.44	167	2	0.06
PF05280	FlhC	Flagellar transcriptional activator (FlhC)	0.19	116	0.00	4.12	118	3	0.07
PF05321	HHA	Haemolysin expression modulating protein	0.03	33	0.00	8.72	34	2	0.02
PF05443	ROS_MUCR	ROS/MUCR transcriptional regulator protein	0.16	362	0.00	6.11	416	20	0.10
PF05764	YL1	YL1 nuclear protein	0.08	12	0.00	10.48	41	20	0.01
PF05848	CtsR	Firmicute transcriptional repressor of class III stress genes (CtsR)	−0.04	191	0.00	2.50	202	8	0.14
PF05930	Phage_AlpA	Prophage CP4-57 regulatory protein (AlpA)	0.18	431	0.00	2.74	482	11	0.18
PF06018	CodY	CodY GAF-like domain	0.01	188	0.00	2.85	398	17	0.12
PF06054	CoiA	Competence protein CoiA-like family	0.04	182	0.00	2.81	198	9	0.12
PF06069	PerC	PerC transcriptional activator	0.02	7	0.00	14.86	9	3	0.01
PF06116	RinB	Transcriptional activator RinB	−0.02	9	0.00	19.41	9	1	0.00
PF06320	GCN5L1	GCN5-like protein 1 (GCN5L1)	−0.03	34	0.00	8.39	214	71	0.02
PF06338	ComK	ComK protein	0.01	93	0.00	4.85	96	4	0.05
PF06769	YoeB_toxin	YoeB-like toxin of bacterial type II toxin-antitoxin system	0.10	350	0.00	2.49	356	4	0.18
PF06818	Fez1	Fez1	0.07	399	0.00	2.77	8316	400	0.16
PF06839	zf-GRF	GRF zinc finger	0.05	4	0.00	28.82	7	4	0.00
PF06923	GutM	Glucitol operon activator protein (GutM)	0.02	76	0.00	4.71	81	6	0.05
PF06943	zf-LSD1	LSD1 zinc finger	0.02	5	0.00	16.28	16	6	0.00
PF07180	CaiF_GrlA	CaiF/GrlA transcriptional regulator	0.00	6	0.00	17.17	9	3	0.00
PF07417	Crl	Transcriptional regulator Crl	0.08	31	0.00	6.48	31	1	0.02
PF07704	PSK_trans_fac	Rv0623-like transcription factor	0.24	142	0.00	4.38	293	16	0.07
PF07716	bZIP_2	Basic region leucine zipper	0.00	35	0.00	7.38	901	152	0.02
PF07750	GcrA	GcrA cell cycle regulator	0.19	211	0.00	3.58	232	12	0.10
PF07764	Omega_Repress	Omega Transcriptional Repressor	-0.03	4	0.00	18.21	4	1	0.00
PF07804	HipA_C	HipA-like C-terminal domain	0.29	814	0.00	1.85	1436	10	0.32
PF07848	PaaX	PaaX-like protein	0.48	272	0.00	2.68	530	19	0.15
PF07879	PHB_acc_N	PHB/PHA accumulation regulator DNA-binding domain	0.31	251	0.00	2.13	553	17	0.18
PF08220	HTH_DeoR	DeoR-like helix-turn-helix domain	0.45	2567	0.01	1.35	5238	87	0.60
PF08222	HTH_CodY	CodY helix-turn-helix domain	−0.01	173	0.00	2.76	340	3	0.12
PF08270	PRD_Mga	M protein trans-acting positive regulator (MGA) PRD domain	−0.06	20	0.00	10.59	57	5	0.01
PF08279	HTH_11	HTH domain	0.35	3290	0.01	1.36	8331	183	0.72
PF08280	HTH_Mga	M protein trans-acting positive regulator (MGA) HTH domain	−0.02	98	0.00	7.17	325	13	0.04
PF08299	Bac_DnaA_C	Bacterial dnaA protein helix-turn-helix	0.20	1385	0.01	0.56	3716	23	0.90
PF09278	MerR-DNA-bind	MerR, DNA binding	0.44	981	0.00	1.75	1988	39	0.35
PF09339	HTH_IclR	IclR helix-turn-helix domain	0.56	4383	0.02	1.85	8834	158	0.58
PF11046	HycA_repressor	Transcriptional repressor of hyc and hyp operons	0.06	5	0.00	16.28	5	1	0.00
PF12324	HTH_15	Helix-turn-helix domain of alkylmercury lyase	0.17	42	0.00	7.61	83	20	0.03
PF12431	CitT	Transcriptional regulator	0.17	82	0.00	5.06	186	8	0.05
PF12793	SgrR_N	Sugar transport-related sRNA regulator N-term	0.14	102	0.00	5.68	215	5	0.04
PF12833	HTH_18	Helix-turn-helix domain	0.62	16065	0.07	1.63	32766	236	0.75
PF13384	HTH_23	Homeodomain-like domain	0.37	2261	0.01	1.55	4836	274	0.63
PF13404	HTH_AsnC-type	AsnC-type helix-turn-helix domain	0.59	2324	0.01	1.68	4460	88	0.52
PF13411	MerR_1	MerR HTH family regulatory protein	0.64	5468	0.02	1.23	7255	206	0.82
PF13412	HTH_24	Winged helix-turn-helix DNA-binding	0.41	4567	0.02	1.12	8874	260	0.81
PF13413	HTH_25	Helix-turn-helix domain	0.23	817	0.00	1.05	1559	60	0.54
PF13545	HTH_Crp_2	Crp-like helix-turn-helix domain	0.57	4228	0.02	1.02	8248	122	0.82
PF13556	HTH_30	PucR C-terminal helix-turn-helix domain	0.47	2065	0.01	2.50	3180	69	0.36
PF13693	HTH_35	Winged helix-turn-helix DNA-binding	0.04	111	0.00	6.64	135	17	0.05
PF13936	HTH_38	Helix-turn-helix domain	0.13	1350	0.01	2.40	2872	165	0.40
PF14549	P22_Cro	DNA-binding transcriptional regulator Cro	0.06	41	0.00	6.64	51	10	0.03
PF14850	Pro_dh-DNA_bdg	DNA-binding domain of Proline dehydrogenase	0.24	315	0.00	1.87	967	4	0.23
PF15723	MqsR_toxin	Motility quorum-sensing regulator, toxin of MqsA	0.11	72	0.00	4.54	103	7	0.05
PF15731	MqsA_antitoxin	Antitoxin component of bacterial toxin-antitoxin system, MqsA	0.06	199	0.00	3.13	271	20	0.12
PF15943	YdaS_antitoxin	Putative antitoxin of bacterial toxin-antitoxin system, YdaS/YdaT	0.08	171	0.00	3.87	231	29	0.09

## Supplementary Materials

The following are available online at https://www.mdpi.com/2073-4425/11/1/52/s1, Table S1: Description of all protein families identified in the bacterial and archaea genomes.
